# Biomass and Its Allocation in Relation to Temperature, Precipitation, and Soil Nutrients in Inner Mongolia Grasslands, China

**DOI:** 10.1371/journal.pone.0069561

**Published:** 2013-07-25

**Authors:** Muyi Kang, Cheng Dai, Wenyao Ji, Yuan Jiang, Zhiyou Yuan, Han Y. H. Chen

**Affiliations:** 1 State Key Laboratory of Earth Surface Processes and Resource Ecology, College of Resources Science and Technology, Beijing Normal University, Beijing, China; 2 Faculty of Natural Resources Management, Lakehead University, Thunder Bay, Ontario, Canada; 3 College of Forestry and Gardening, Anhui Agricultural University, Hefei, China; University of Alberta, Canada

## Abstract

**Aim:**

Understanding and predicting ecosystem functioning such as biomass accumulation requires an accurate assessment of large-scale patterns of biomass distribution and partitioning in relation to climatic and soil environments.

**Methods:**

We sampled above- and belowground biomass from 26 sites spanning 1500 km in Inner Mongolian grasslands, compared the difference in aboveground, belowground biomass and below-aboveground biomass ratio (AGB, BGB, and B/A, respectively) among meadow steppe, typical steppe, and desert steppe types. The relationships between AGB, BGB, B/A and climatic and soil environments were then examined.

**Results:**

We found that AGB and BGB differed significantly among three types of grasslands while B/A did not differ. Structural equation model analyses indicated that mean annual precipitation was the strongest positive driver for AGB and BGB. AGB was also positively associated with soil organic carbon, whereas B/A was positively associated with total soil nitrogen.

**Conclusions:**

These results indicated that precipitation positively influence plant production in Inner Mongolian grasslands. Contrary to the prediction from the optimal partitioning hypothesis, biomass allocation to belowground increased with soil total nitrogen, suggesting that more productive sites may increase belowground allocation as an adaptive strategy to potentially high fire frequencies.

## Introduction

Grassland ecosystems, accounting for about a quarter of global land area and 10 percent of global carbon storage, play an important role in regulating the global carbon cycle [1], [2]. The area of Chinese grasslands is approximately 1.18×10^8^ hectares [3], accounting for approximately 8 percent of total area of global grasslands [4]. Of the Eurasian grasslands, Inner Mongolian grasslands are the vastest.

Previous studies have shown that grassland biomass is influenced by both climate and soil characteristics [5]. However, studies about the influence of climate on grassland biomass have so far focused mostly on aboveground biomass (AGB). By contrast, belowground biomass (BGB), which accounts for a substantially higher portion of total ecosystem biomass in grasslands [6], [7] is insufficiently studied [8], largely because of lack of a simple and efficient method to accurately determine BGB [7], [9]. In particular, our understanding of soil influence on BGB and belowground biomass allocation is limited. For Chinese temperate grasslands, several studies indicate that biomass is strongly influenced by precipitation [10], [11]. However, the response of biomass and its allocation to multiple environmental drivers, especially soil conditions, remains unclear.

Biomass allocation, typically assessed by belowground to aboveground biomass ratio (B/A) at the ecosystem level or shoot to root ratio at the individual plant level [12], reflects the strategy of plants or ecosystems to partition photosynthate in belowground and aboveground tissues [8]. Plants or ecosystems typically increase B/A ratio to take up limited belowground resources under nutrient-poor and/or water deficit conditions [13]–[16]. The idea has received support from global meta-analyses that consider a wide range of terrestrial ecosystems, where vegetation types co-vary with resource availability [12], [17]. However, the B/A ratio has been reported to be insensitive to climatic variations in Chinese grasslands [10], [11].

In this study, we examined the patterns of AGB, BGB and B/A along temperature and precipitation gradients in Inner Mongolian grasslands. We further examined whether patterns of AGB, BGB and B/A are associated with soil resource availability along temperature and precipitation gradients using structural equation models. To understand casual relationships between AGB, BGB or B/A and climate and soil variables, we used structural equation models to account for direct, indirect, and total effects of one variable on another [18]–[22].

## Materials and Methods

### Study Area and Sites

**Figure 1:**
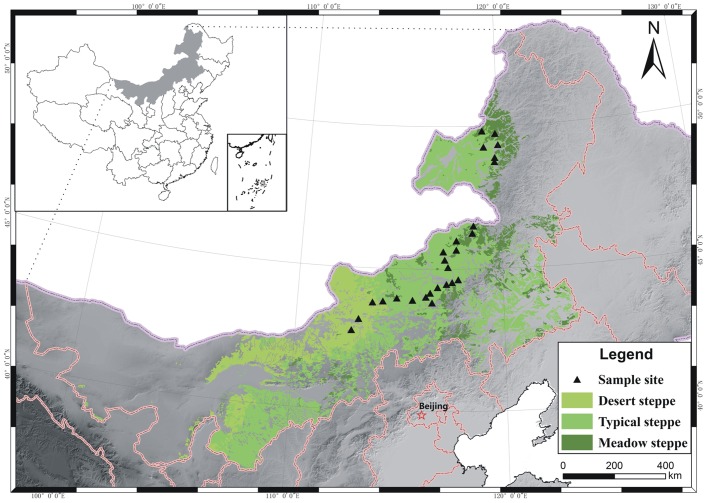
The distribution of sample sites in Inner Mongolia, China.

The study area is located in the middle and east of Inner Mongolia, the core part of the Inner Mongolian grasslands. The longitude extends from 112°40′ E to 121°10′ E and the latitude from 42°48′ N to 49°20′ N (Fig. 1). No specific permissions are required for our conducting field survey in this area, since land in China belongs to the public and our field studies did not involve any endangered or protected species within. The transect line extended from the Ulan Qab Plateau, through the Xilin Gol Grassland and the Hulun Buir Prairie to Horqin Grassland. This area is dominated by arid and semi-arid temperate continental to continental monsoon climate, which is characterized by cold, long winters and hot, rainy, and short summers. From southwest to northeast, mean annual temperature (MAT) ranged from −2.4 to 5.1°C, and mean annual precipitation (MAP) ranged from 176 to 376 mm. The soil types in these sites included sierozem, brown calcic soil, kastanozem (chestnut soil), chernozem, dark brown soil, and black soil [23]. According to China’s vegetation classification system, the grassland was divided into three types: desert steppe, typical steppe, and meadow steppe [24]. The desert steppe was dominated by *Stipa glareosa* and *S. klemenzii*. The dominant species in the typical steppe were *S. grandis*, *S. krylovii,* and *Artemisia frigida*, whereas in the meadow steppe *S. Baicalensis*, *Leymus chinensis* and *Filifolium sibiricum* were dominant species.

### Data Collection

Samples were collected in August 2010, when grassland biomass was at its peak in Inner Mongolian grasslands. In order to sample a wide range of climates and soil conditions, we selected 26 sites from Sonid Right Banner in the southwest to Hulun Buir in the northeast using a transect with approximately 50 km apart between adjacent sites. The distance between the Hulun Buir Prairie and the Xilin Gol Steppe sites, separated by the Greater Khingan Range was about 200 km. The selected sites were characterized by flat relief, protected for at least 10 years by fences constructed by local governments to monitor long-term ecosystem dynamics without human disturbances such as grazing and mowing. In each site, three plots with an area 15×15 m^2^ were randomly assigned with a distance of at least 20 m between the two adjacent plots.

#### Aboveground Biomass (AGB)

Four 0.5×0.5 m^2^ quadrats were randomly placed within each plot. The constituent species were recorded, and their height and cover were respectively measured. All plants were clipped at the soil surface and taken to the laboratory. After removing sand and gravel, the biomass samples were oven-dried at 65°C to a constant mass and then weighed.

#### Belowground Biomass (BGB)

Roots were extracted with a soil corer (8 cm in diameter) at 10 cm intervals to a depth of 30 cm using a power auger, a similar method used by Brassard et al. [25]. In each plot, four cores were taken, with a total number of 12 cores in each site. The core samples were soaked fully in the water to remove soil. After washing through a 0.2 mm mesh sieve, roots were put in paper bags, taken to the laboratory, oven-dried at 65°C to a constant mass and then weighed.

#### Soil characteristics

In each site, three 30 cm-deep soil profiles were excavated, each separated into three layers with a depth of 10 cm to collect soil samples. After being air-dried in a cool, well-ventilated place, the samples were passed through a 1 mm sieve and roots were removed. Soil organic carbon (SOC) and total nitrogen (TN) were measured using Vario EI elemental analyzer (Elementar Company Inc., Hanau, Germany). Potentiometric method was used to measure pH. Soil characteristics were averaged for each site in final analysis.

#### Climate data

Climatic variables including mean annual temperature (MAT) and mean annual precipitation (MAP) from 1960 to 2009 were obtained from 58 meteorological stations located in or around sample sites from China Meteorological Administration. The climate data were interpolated by Kriging interpolation method using ArcGIS10.0 (ESRI Company Inc., Redlands, California, USA) for each of our 26 sample plots according to their geographic coordinates (latitude, longitude, and elevation).

### Data Analysis

Differences in AGB, BGB, total biomass (i.e., sum of AGB and BGB), and B/A among three grassland types were tested by one-way analysis of variance (ANOVA), followed by Tukey’s post hoc test when grassland type was significant. The assumption of normal distribution was met according to Shapiro-Wilk test. A logarithmic transformation was needed for AGB, BGB, and TB to meet the assumptions of normality and homogenous variances. Because inherently complex causal connections exist among environmental variables in natural environments [18]–[22], we developed structural equation models (SEM) to determine the direct, indirect, and total effects of environmental variables on AGB, BGB, or B/A. For each SEM model, we hypothesized paths between endogenous variable (i.e., AGB, BGB or B/A) and exogenous variables (i.e., MAT, MAP, TN, SOC, and pH) and casual paths of climatic variables on soil variables. We specified correlations between MAT and MAP and among soil variables (TN, SOC, and pH). To better meet SEM’s assumption of linearity between dependent and independent variables, we applied logarithmic transformation to both dependent and independent variables. The linearity assumption was verified by plotting the residuals after fitting linear regression between the log-transformed dependent variable and each of log-transformed independent variable [26]. Since the values in MAT were smaller than zero in a few sites, all MAT values were transformed by adding 3 to eliminate negative values. Statistical analyses were carried out by using the SPSS 19.0 package (IBM Company Inc., Armonk, New York, USA) and structural equation models were analyzed using AMOS package (expansion pack of SPSS).

## Results

### AGB, BGB and B/A among Grassland Types

**Figure 2:**
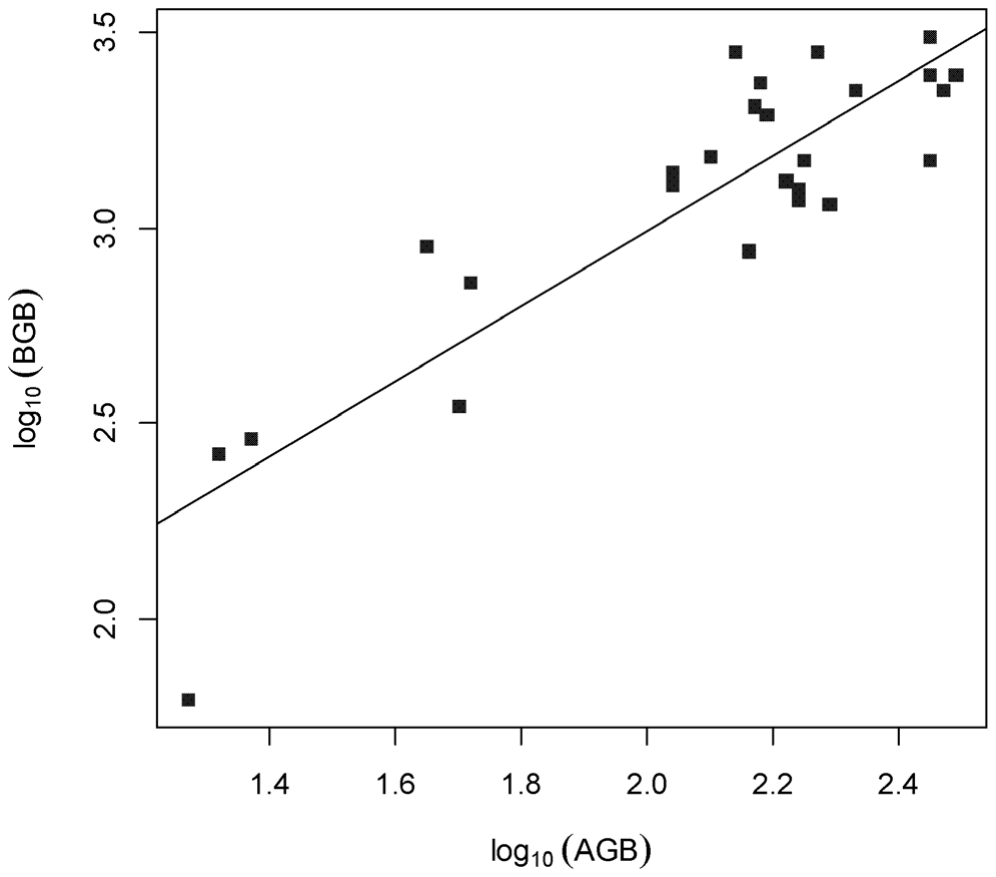
Relationship between aboveground biomass (AGB) and belowground biomass (BGB) (*n = *26). The fitted regression is *y* = 1.064+0964*x* (*R* ^2^ = 0.77, *P*<0.001).

AGB, BGB and TB differed significantly among the three grassland types (Table 1). AGB, BGB and TB decreased from meadow steppe to typical steppe and to desert steppe (*P*<0.001). Mean AGB, BGB and TB of meadow steppe were 228.7, 2511.7, and 2740.5 g·m^−2^, respectively (Table 1). In typical steppe, they were 162.8, 1556.1, and 1718.9 g·m^−2^, respectively (Table 1). In desert steppe, they were 28.5, 240.7, and 269.2 g⋅m^−2^, respectively. On average, AGB, BGB and TB of Inner Mongolia grasslands were 154.8, 1537.5, 1692.3 g·m^−2^, respectively (Table 1). However, B/A ratio, with an average value of 10.6, tended to increase from meadow, typical, to desert steppe (Table 1), but did not differ significantly among the three steppe types (*P* = 0.47). With all data pooled, AGB and BGB were strongly correlated, as the relationship between BGB and AGB (*r* ^2^ = 0.78, *P*<0.001, Fig. 2).

**Table 1:** The aboveground biomass (AGB), belowground biomass (BGB), total biomass (TB) and belowground to aboveground biomass ratio (B/A) among three grassland types in Inner Mongolia (mean ±1 SE).

Grasslandtype	*n*	AGB(g·m^−2^)	BGB(g·m^−2^)	TB(g·m^−2^)	B/A
Meadowsteppe	5	228.7(28.6)^a^	2511.7(203.6)^a^	2740.5(219.0)^a^	11.4(1.2)^a^
Typicalsteppe	17	162.8(17.3)^a^	1556.1(147.5)^b^	1718.9(156.4)^b^	10.8(1.2)^a^
Desertsteppe	4	28.5(7.3)^b^	240.7(62.3)^c^	269.2(67.5)^c^	8.7(2.2)^a^
Total	26	154.8(17.1)	1537.5(167.7)	1692.3(164.2)	10.6(0.8)

Within the same columns, values with different superscripts letters (a, b, c) indicate significant difference (α <0.05) between grassland types.

### Casual Effects of Climate and Soil Variables on AGB, BGB and B/A

**Figure 3:**
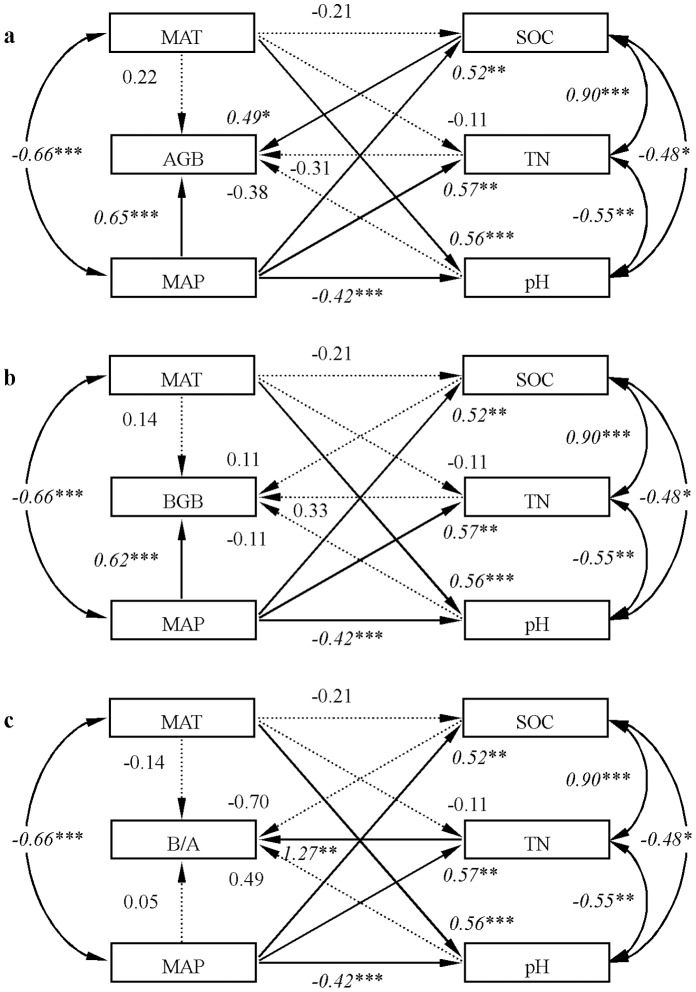
Results of structural equation models for: a) aboveground biomass (AGB), b) belowground biomass (BGB), and c) belowground to aboveground biomass ratio (B/A). Each arrow represents a direct linear causal relationship. The arcs show the correlation between two variables. Values on arrows are path coefficients and on arcs are standardized correlation coefficients. Italic values indicate the coefficients are significant at *P*<0.05 (*), *P*<0.01 (**), and *P*<0.001 (***). The coefficients that are not statistically significant are shown by dashed arrows. All values are log-log transformed.

Climatic variables, MAT and MAP, were negatively correlated (Fig. 3, *r* = −0.66, *P*<0.001). Soil variables were also strongly correlated (Fig. 3). MAP affected all soil variables: direct effects of MAP on SOC, TN, and pH scored at 0.52 (*P*<0.01), 0.57, (*P*<0.01); and −0.42 (*P*<0.001), respectively (Table 2, Fig. 3). MAT also had direct effects on pH (path coefficient = 0.56, *P*<0.001) (Table 2). Additionally, there was a strong positive correlation between SOC and TN (*r* = 0.90, *P*<0.001), and significant negative correlations were found between pH and SOC (*r* = −0.48, *P*<0.05) and between pH and TN (*r* = −0.55, *P*<0.01). Climatic variables strongly influenced soil variables (Fig. 3).

**Table 2:** Standardized direct effect, indirect effect, and total effect of mean annual temperature (MAT), mean annual precipitation (MAP), soil organic carbon (SOC), soil total nitrogen, and soil pH on aboveground biomass (AGB), belowground biomass (BGB), and below- to aboveground biomass ratio (B/A).

Endogenous variable	Exogenous variable	Direct effect	Indirect effect	Total effect
AGB	MAT	0.22	−0.28	−0.06
	MAP	0.65***	0.24	0.89
	SOC	0.49*	0	0.49
	TN	−0.31	0	−0.31
	pH	−0.38	0	−0.38
BGB	MAT	0.14	−0.12	0.02
	MAP	0.62***	0.29	0.91
	SOC	0.11	0	0.11
	TN	0.33	0	0.33
	pH	−0.11	0	−0.11
B/A	MAT	−0.14	0.28	0.14
	MAP	0.05	0.16	0.21
	SOC	−0.70	0	0.70
	TN	1.27**	0	1.27
	pH	0.49	0	0.49

Significant effects are indicated at *(*P*<0.05), **(*P*<0.01), and ***(*P*<0.001).

Note: full table is presented in Table S1, Table S2 and Table S3 in File S1.

MAP had the strongest positive effects on both AGB and BGB among all predictors (Table 2, Figs. 3a, b), whereas no significant effect of MAT on AGB or BGB was found. AGB and BGB responded similarly to MAP. Standardized total effect of MAP on AGB was 0.89, consisting of direct effect (path coefficient = 0.65, *P*<0.001) and indirect effects through soil variables (path coefficient = 0.89–0.65 = 0.24). The total effect of MAP on BGB was 0.91, consisting of direct effect (path coefficient = 0.62, *P*<0.001) and indirect effects through soil variables (path coefficient = 0.91–0.62 = 0.29). Among soil variables, only SOC had a significant positive effect on AGB (path coefficient = 0.49, *P*<0.05) (Fig. 3a). The structural equation models explained 89% and 91% of the variation in AGB and BGB, respectively.

The structural equation model explained 37% of the variation in B/A (Fig. 3c). Among soil variables, only TN had a significant positive effect on B/A (Standardized direct effect of TN on B/A, path coefficient = 1.27, *P*<0.01). However, neither direct nor indirect effects of MAT and MAP on B/A were significant.

## Discussion

The present study is, to our knowledge, the first to demonstrate that both climatic and soil characteristics simultaneously affect both above- and belowground biomass in Inner Mongolian grasslands, China. Furthermore, we show that biomass allocation to below- and aboveground did not change along the climate gradient in Inner Mongolian grasslands, but positively associated with total nitrogen availability along the studied environmental gradients.

### Variation in Biomass and its Allocation

The mean AGB and BGB of our study area (154.84 g m^−2^ and 1537.49 g m^−2^, respectively) were higher than those reported by Yang et al. [11] in the same study area (AGB = 116.6 g m^−2^, BGB = 553.3 g m^−2^). The higher biomass observed in this study than that by Yang et al. [11] may be attributed to the fact that our sample sites were protected from human disturbances such as grazing and mowing, and also that the proportion of desert sites in our study was smaller. Our mean BGB was similar to, but our mean AGB was smaller than the respective global averages reported by Jackson et al. [6]. Consequently, our B/A (10.6) was higher than the global mean [3.7 reported by Jackson et al. [6] and 4.5 reported by Mokany et al. [12]] and the overall mean of Chinese grasslands [5.7 reported by Yang et al. [11]]. However, the B/A in our study was within the top range of those reported by Jackson et al. [6] and Mokany et al. [12], and was lower than the values reported by a previous study in Inner Mongolia grasslands [10].

### Relationships of Biomass and Environmental Factors

Our structural equation models explained about 90% of the variation in AGB and BGB, supporting the idea that water, temperature, and soil are the main environmental factors influencing grassland biomass [27]. Our results show that precipitation had the strongest effect on AGB and BGB. Although temperature, a surrogate of energy available to plants, is positively associated with production at a global scale [28], our results show no significant influence of temperature on AGB and BGB in our study area, indicating that there is a tradeoff effect between temperature and precipitation on plant growth. Our findings reinforce that precipitation is a major limiting factor influencing biomass in arid and semi-arid grassland ecosystems [29], [30].

Soil nutrients, in particular nitrogen, have been found to be a limiting factor for both above- and belowground production in most terrestrial ecosystems [31]–[33]. Nevertheless, others have reported limited or no influence of soil nitrogen on ecosystem production [34]–[36]. It appears that the different responses of plants to increasing nitrogen content are a result of different nitrogen deficiency levels of the local systems. However, when TN and SOC were treated as casual predictors in our structural equation models, only SOC had a positive effect on aboveground biomass.

Our results show support for the notion that plant growth in natural grasslands is primarily limited by water, resulting in high amounts of biomass allocation to belowground in order to capture these resources [37], [38]. The effect of nitrogen availability on grassland biomass is conditional to water availability [39]. Dry regions with low water availability such as Inner Mongolian grasslands may also prevent nitrogen from becoming available, as nutrients are not in solution [40].

### Factors Influencing Biomass Allocation

We showed a complex web of causations for B/A in Inner Mongolian grasslands. However, our results indicated that B/A did not respond to climatic gradients, but responded positively to soil nitrogen. These results are in disagreement with the prediction from the optimal partitioning hypothesis [41]. This disagreement can be resulted from several reasons. First, despite the large spatial scale in our study and other similar studies in dry regions [10], [11], [42], the climatic variations in these studies represent only a small fraction of the global climatic variations that are considered in global meta-analyses [12], [17]. Therefore, the differences in ecosystem-specific results and global syntheses reflect different ecological scales. Second, Mccarthy and Enquist [43] suggest that the patterns of optimal partitioning can vary from species to species, obscuring large-scale patterns such as our study. The positive association between B/A and soil nitrogen may reflect a shift in plant composition along the soil nitrogen gradient [44]. Third, mean annual precipitation and temperature are negatively correlated along the climate gradients in our study. The biomass in dry and warm sites may not be sufficient to support frequent fires, whereas sites with more precipitation may have high fire frequencies due to more frequent lightning ignitions and available biomass fuels. As hypothesized by Bhattachan et al. [42], high allocation to belowground due to the need for additional storage as the risk of fire increases may be an important life-history strategy for sites with high percipitation and high fire frequencies.

### Conclusions

Our results show that precipitation strongly affects aboveground and belowground biomass in semi-arid ecosystems. Moreover, our results show that belowground to aboveground biomass ratio is positively associated with soil total nitrogen, but this ratio is not related to climatic variables. Future work shall attempt to partition the influences of environmental variations and vegetation types on biomass allocation.

## Supporting Information

File S1 Table S1, Total, direct, and indirect effects of mean annual temperature (MAT), mean annual precipitation (MAP), soil organic carbon (SOC), soil total nitrogen, and soil pH on aboveground biomass (AGB), belowground biomass (BGB), and below- to aboveground biomass ratio (B/A); Table S2, Effects of MAT and MAP on soil variables. Abbreviations are same as in Table S1; Table S3, Correlations among exogenous variables. Abbreviations are same as in Table S1.
(DOC)
