# Supplementary material for: Biomass and Its Allocation in Relation to Temperature, Precipitation, and Soil Nutrients in Inner Mongolia Grasslands, China
Source: PLoS One. 2013 Jul 25;8(7):e69561. doi: 10.1371/journal.pone.0069561 (PMC3723834; doi:10.1371/journal.pone.0069561)
Supplement: File S1 — Table S1, Total, direct, and indirect effects of mean annual temperature (MAT), mean annual precipitation (MAP), soil organic carbon (SOC), soil total nitrogen, and soil pH on aboveground biomass (AGB), belowground biomass (BGB), and below- to aboveground biomass ratio (B/A); Table S2, Effects of MAT and MAP on soil variables. Abbreviations are same as in Table S1; Table S3, Correlations among exogenous variables. Abbreviations are same as in Table S1. (DOC) [file pone.0069561.s001.doc]

# Supporting information S1

# Table S1

Total, direct, and indirect effects of mean annual temperature (MAT), mean annual precipitation (MAP), soil organic carbon (SOC), soil total nitrogen, and soil pH on aboveground biomass (AGB), belowground biomass (BGB), and below- to aboveground biomass ratio (B/A).

| Endogenous variable | Exogenous variable | Total effect | Standardized Total Effects | Direct Effects | Standardized Direct Effects | Indirect Effects | Standardized Indirect Effects |
| --- | --- | --- | --- | --- | --- | --- | --- |
| AGB | MAT | -0.072 | -0.058 | 0.276 | 0.223 | -0.348 | -0.281 |
|  | MAP | 3.349 | 0.886 | 2.460 | 0.651 | 0.889 | 0.235 |
|  | SOC | 0.911 | 0.488 | 0.911 | 0.488 | 0 | 0 |
|  | TN | -0.550 | -0.308 | -0.550 | -0.308 | 0 | 0 |
|  | pH | -3.951 | -0.375 | -3.951 | -0.375 | 0 | 0 |
| BGB | MAT | 0.019 | 0.014 | 0.184 | 0.135 | -0.165 | -0.121 |
|  | MAP | 3.760 | 0.906 | 2.562 | 0.617 | 1.198 | 0.289 |
|  | SOC | 0.230 | 0.112 | 0.230 | 0.112 | 0 | 0 |
|  | TN | 0.637 | 0.325 | 0.637 | 0.325 | 0 | 0 |
|  | pH | -1.278 | -0.110 | -1.278 | -0.110 | 0 | 0 |
| B/A | MAT | 0.091 | 0.140 | -0.092 | -0.142 | 0.183 | 0.282 |
|  | MAP | 0.411 | 0.208 | 0.103 | 0.052 | 0.309 | 0.156 |
|  | SOC | -0.681 | -0.699 | -0.681 | -0.699 | 0 | 0 |
|  | TN | 1.188 | 1.272 | 1.188 | 1.272 | 0 | 0 |
|  | pH | 2.673 | 0.485 | 2.673 | 0.485 | 0 | 0 |

# Table S2

Effects of MAT and MAP on soil variables. Abbreviations are same as in Supplementary Information Table S1.

| Climatic variable | Soil variable | Total effect | Standardized Total Effects | Direct Effects | Standardized Direct Effects | Indirect Effects | Standardized Indirect Effects |
| --- | --- | --- | --- | --- | --- | --- | --- |
| MAT | SOC | -0.141 | -0.212 | -0.141 | -0.212 | 0.000 | 0.000 |
| MAP |  | 1.052 | 0.519 | 1.052 | 0.519 | 0.000 | 0.000 |
| MAT | TN | -0.076 | -0.110 | -0.076 | -0.110 | 0.000 | 0.000 |
| MAP |  | 1.200 | 0.568 | 1.200 | 0.568 | 0.000 | 0.000 |
| MAT | pH | 0.066 | 0.564 | 0.066 | 0.564 | 0.000 | 0.000 |
| MAP |  | -0.150 | -0.418 | -0.150 | -0.418 | 0.000 | 0.000 |

# Table S3

Correlations among exogenous variables. Abbreviations are same as in Supplementary Information Table S1.

| Exogenous variable | MAT | MAP | SOC | TN | pH |
| --- | --- | --- | --- | --- | --- |
| MAT | 1.000 |  |  |  |  |
| MAP | -0.657 | 1.000 |  |  |  |
| SOC | -0.553 | 0.658 | 1.000 |  |  |
| TN | -0.483 | 0.640 | 0.898 | 1.000 |  |
| pH | 0.839 | -0.789 | -0.482 | -0.551 | 1.000 |
